# Proteomic Characterization of Dry Blood Spots of Healthy Women During Simulation the Microgravity Effects Using Dry Immersion

**DOI:** 10.3389/fphys.2021.753291

**Published:** 2022-01-11

**Authors:** Daria N. Kashirina, Alexander G. Brzhozovskiy, Wen Sun, Ludmila Kh. Pastushkova, Olga V. Popova, Vasiliy B. Rusanov, Evgeny N. Nikolaev, Irina M. Larina, Alexey S. Kononikhin

**Affiliations:** ^1^Institute of Biomedical Problems – Russian Federation State Scientific Research Center, Russian Academy of Sciences, Moscow, Russia; ^2^CDISE, Skolkovo Institute of Science and Technology, Moscow, Russia

**Keywords:** proteomics, dried blood spots, dry immersion, timsTOF pro mass spectrometry, red blood cells

## Introduction

Dry immersion (DI) is one of the most widely used terrestrial microgravity models. Dry immersion accurately and quickly reproduces most of the physiological effects of the early period of space flights (Tomilovskaya et al., 2019). The model simulates such factors of space flight as lack of support, mechanical and axial unloading, lack of physical activity. Almost complete immersion in water makes it possible to simulate cardiovascular, musculoskeletal, and other effects of microgravity (Navasiolava et al., 2010). There is a 17% loss of plasma volume observed after 2 days of DI and which is comparable to post-flight observation (Treffel et al., 2017). Dry immersion experiment used as microgravity model leads to the body fluid media displacement as in space flight, which is associated with a uniform compression of the subject's body. Body fluid media displacement leads to a decrease in heart rate within a few hours after immersion in water. At the same time, the heart rate is reduced by five beats per minute, and the blood pressure is reduced by 5 mm Hg in the first 4 h (Navasiolava et al., 2010). Similar changes in heart size and stroke volume are observed both in DI and in space flight. Dry immersion also causes muscle wasting and changes in the structure of the musculoskeletal system similar in nature and timing of development to those changes that occur in space flight. This is mainly due to the absence of gravitational stress exerted on the object of study (Treffel et al., 2017). Although the physiological response of the organism to DI conditions is similar to that during the space flight there are some differences that need to be studied on molecular level.

Regulatory and metabolic changes occurring in DI are reflected in the protein composition of body fluids. In the blood, these changes were previously fragmentarily studied. Thus, in an experiment with 5-day DI with 20 young healthy men, an increase in the level of unconjugated bilirubin and myoglobin in serum was revealed, which confirmed that DI could promote hemolysis and myolysis. Increases in hepcidin, ferritin, and haptoglobin have also been shown, which may be associated with increased serum iron levels (Nay et al., 2020). Previously, a 7-day experiment simulating unsupported conditions was conducted with participation of five healthy male volunteers who were not exposed to any additional influences. The results obtained by the method of two-dimensional electrophoresis showed a change in the concentration of proteins of the hemostasis and complement systems: a decrease in the concentration of α-, β-fibrinogen, factor C4B complement, and serum amyloid P (Trifonova et al., 2010). On the last day of 7-day DI a significant decrease in fragments of fibrinopeptide A and peptide activator of coagulation factor XIII was found both in the control group and in the group with prophylaxis. A lower concentration of these proteins could indicate a decrease in the fibrinolytic activity of the blood and shifts in the hemostatic system during immersion (Pakharukova et al., 2011). At the same time, on the 7th day after the end of the experiment, a significant increase in the blood level of apolipoproteins A–I, A–IV, and also E after DI was observed which may indicate a changes in the functioning of lipid transport during the adaptation period (Trifonova et al., 2010). In the course of 5-day DI with 14 men participants, an increase in the peaks of proteins C3 and C4 of the complement system, high molecular weight kininogen and fibrinogen was found which also may confirm the involvement of the hemostasis and the complement systems in the body's response to conditions of DI (Pastushkova et al., 2012). Comparative analysis of changes in the protein composition of blood after a real space flight and model experiments (21 days of head-down bed rest and 21 days of DI) revealed nine common proteins (A1BG, A2M, SERPINA1, SERPINA3, SERPING1, SERPINC1, HP, CFB, TF) which change the level after landing and in the ground experiments. These proteins made it possible to identify processes influenced by microgravity, including hemostasis, platelet degranulation, and protein metabolism (Brzhozovskiy et al., 2019).

Proteomics methods have not yet become widespread in the study of the effects of extreme conditions on the human body. Analysis of human blood and urine proteome is challenging because almost all proteins synthesized in the human body get into the plasma and the dynamic range of proteins concentrations is quite large. The next challenge is data analysis and interpretation since most of proteins are multifunctional and participate in many biological pathways forming a complex network of molecular interactions. Nevertheless, proteomic methods have made it possible to determine proteins that respond to a complex set of DI conditions. System analysis of the proteomic data and biological pathways made it possible to clarify the molecular mechanisms of changes caused by DI in various physiological systems, including the cardiovascular system. Proteome changes in biological fluids may indicate the processes of atherogenesis, neoangiogenesis, changes in cell adhesion, metabolism of the extracellular matrix, which ultimately may affect the function of the heart and blood vessels (Rusanov et al., 2020). In addition to confirming the already known effects of microgravity, proteomic analysis is able to reveal new effects that appear at the molecular level but have not yet been considered.

Proteomics methods are becoming more powerful and sensitive tools for studying the protein composition in different biological samples. Dried blood spot (DBS) micro-sampling technique has already been found to be useful for both population-wide screening and in research studies. Detecting up to 700 proteins in DBS sample has become quite possible which expand the availability for detecting and quantifying proteins (Eshghi et al., 2020). A deep blood proteome analysis can facilitate the discovery of new data and mechanisms of human adaptive response to DI conditions.

Thus, the aim of the study was to apply blood proteome analysis with DBS micro-sampling technique to study on the molecular level the physiological response to the DI conditions and provide new information about previously unknown mechanisms of the adaptive processes. In this work an untargeted proteomic analysis of DBS samples collected from six healthy young women during 3 days DI experiment was performed. As far as we know this is the first proteomic study of the female reaction to simulated weightlessness. The adaptive reactions of the body were monitored on a daily basis during DI experiment.

## Materials and Methods

### Dry Immersion Experiment Design

Six healthy young women (age 30.17 ± 5.5 years) participated in the experiment with 3-day DI. The study involving women was carried out for the first time. The experiment was organized by the State Research Center of the Russian Federation—IBMP RAS, Moscow, and carried out at the “dry immersion” stand on the territory of the IBMP RAS. The stand is a part of the unique scientific installation “Medical and technical complex for the development of innovative technologies of space biomedicine in the interests of ensuring orbital and interplanetary flights, as well as the development of practical health care.” The bath was modified, which made it possible to automate many technical systems while strictly preserving all its functional qualities.

All the volunteers were found healthy by the medical expert commission and admitted to conduct the tests. Previously, the research procedures and methods were reviewed and approved by the Commission on Biomedical Ethics at the IBMP RAS. All subjects signed written informed consent. To simulate the physiological effects of microgravity, the subjects were immersed in water in a prone position to the level of the upper third of the shoulder (water *t* = 33–34°C), but did not come into contact with it, being separated from the water by a waterproof fabric, which was fixed to the sides in such a way that it remained free, not taut. During the DI, volunteers were not exposed to any additional influences aimed at preventing the development of adaptive shifts in physiological systems. Every evening the subject was raised from the bath for an average of 15–20 min for hygiene procedures, most of which were carried out in the subject's lying position. The diet was balanced. Water consumption was free. In order to avoid various influences on the studied parameters of the hormonal background of the normal menstrual cycle, the participants were synchronized by the phase of the cycle for the period of the beginning of the study (proliferative phase).

### Capillary Blood Sampling

Capillary blood was obtained by puncturing the phalanx of the fourth finger with an automatic scarifier. Using an automatic pipette, 40 μl of blood was taken and placed on filter paper for drying. After collection, the blood stains were dried on filters at ambient temperature (19–26°C) for 2–3 h with minimal exposure to sunlight, and then placed in a zip-lock bag. The filters were stored at a temperature of −20°C before further sample preparation for LC-MS/MS analysis.

### Sample Preparation

The DBS was excised and placed in a 1.5 ml polypropylene Eppendorf tube. Proteins were extracted in 1 ml of a solution of 25 mM ammonium bicarbonate, 1% sodium deoxycholate, and 5 mM TCEP (tris (2-carboxyethyl) phosphine hydrochloride) (Thermo Scientific) at 60°C with vortexing at 1,000 rpm (Thermomixer, Eppendorf) for 1 h.

The sample preparation method included reduction with 0.1 M dithiothreitol in 0.1 M Tris buffer (pH 8.5) containing 8 M urea for 30 min at 47°C, as well as alkylation with 0.05 M iodoacetate and incubation for 30 min in the dark at room temperature. Then precipitation was carried out with five volumes of acetone in the presence of 0.1% TFA at −20°C overnight.

The sample preparation procedure was completed by trypsinolysis in 0.05 M ammonium bicarbonate buffer. To the precipitated mixture of proteins were added 100 μl of the buffer and 2 μl of trypsin solution at a concentration of 1 μg/μl in 50 mM acetic acid. Incubation was carried out overnight in a thermomixer at 37°C with shaking at 750 rpm. Then, 1 μl of a 10% aqueous formic acid solution was added to inactivate trypsin and precipitate DOC. The sample was centrifuged at 21,000 g for 10 min, and 20 μl of the supernatant was transferred to a new tube. At this stage, the sample was ready for mass spectrometric analysis.

### LC–MS/MS Proteomic Analysis

The resulted tryptic peptide mixture was analyzed using liquid chromatography–mass spectrometry method based on a nano-HPLC Dionex Ultimate3000 system (Thermo Fisher Scientific, USA) and a timsTOF Pro (Bruker Daltonics, USA) mass spectrometer. A packed emitter column (C18, 25 cm × 75 μm × 1.6 μm) (Ion Optics, Parkville, Australia) was used to separate peptides at a flow rate of 400 nl/min by gradient elution from 4 to 90% of phase B during 40 min. Mobile phase A consisted of 0.1% formic acid in water and mobile phase B consisted of 0.1% formic acid in acetonitrile.

Mass spectrometric analysis was performed using the parallel accumulation serial fragmentation (PASEF) acquisition method (Meier et al., 2018). An electrospray ionization (ESI) source was operated at 1,500 V capillary voltage, 500 V end plate offset, and 3.0 L/min of dry gas at temperature of 180°C. The measurements were carried out in the m/z range from 100 to 1,700 Th. The ion mobility was in the range from 0.60 to 1.60 V s/cm^2^. The total cycle time was 1.88 s and the number of PASEF MS/MS scans was set to 10.

### Data Analysis

The LC-MS/MS data obtained were analyzed using PEAKS Studio 8.5. The database search for protein/peptides identification was performed against the SwissProt database with the following parameters: parent mass error tolerance–20 ppm; fragment mass error tolerance −0.03 Da; enzyme—trypsin; missed cleavages–3; fixed modifications—Carbamidomethyl (C); variable modifications—Oxidation (M), Acetylation (N-term). False discovery rate (FDR) threshold was set to 0.01. All the LC-MS/MS and proteomics results have been deposited to the ProteomeXchange Consortium via the PRIDE (Vizcaino et al., 2016) partner repository with the dataset identifier PXD027654 and 10.6019/PXD027654. Proteins quantification was carried out using label-free method using normalized intensities of corresponding tryptic peptides across all samples and represented by a normalized intensity profile (sample area units from PEAKS) that was extracted from LC-MS/MS data.

### Statistical Methods

To identify significant differences in the concentration of proteins between the points of the experiment, analysis of variance (ANOVA, *p*-value <0.05) was used in the Statistica 12 program.

### Bioinformatic Tools

Functional protein annotation was carried out using the String web resource (https://string-db.org).

### Dynamics of Proteomic Changes via Dry Blood Spots

All dry blood spots (DBS) were analyzed with one of the top proteomics methods based on the PASEF acquisition approach implemented on the timsTOF Pro instruments. The approach allowed us to perform deep DBS proteome analysis and to identify ~500–700 proteins in each sample with relative concentrations of each protein. A total of 1,256 proteins were identified and the relative concentrations were determined at least at one of the five experimental points during DI experiment.

Comparison of the relative concentrations of proteins made it possible to reveal the dynamics of changes in the content of proteins in the DBS samples. As a result of ANOVA 24 proteins were identified as significantly different (*p*-value < 0.05) between the time points during DI experiment (Table 1). A *post-hoc* analysis by the Tukey method showed that statistically significant differences in protein concentrations mainly occurs on 2^nd^ and 3^rd^ days of DI relative to the background (2 days before DI). At the same time the proteins with the most significantly changed level at the end of DI experiment and relative to the background were identified. The summary information regarding significantly changed proteins is presented in Table 1 and shows the day of the experiment on which the protein concentration significantly differed from the background values. It is interesting to note that on first day of DI only one protein differed from the background while on the second day 12 proteins differed. However, on the third day some adaptation to the experimental conditions was observed—eight proteins differed. After leaving the experiment on the second day 5 proteins still did not come to background levels. Among these proteins is the proteasome subunit alpha type-2 (PSMA2) which is involved in the proteolytic degradation of most intracellular proteins. It should be noted that proteasomal protein degradation is enhanced including the response to oxidative stress (Kaya and Radhakrishnan, 2020). Concentrations of immunoglobulin heavy constant alpha 1 (IGHA1), profilin-1 (PFN1) which at low concentrations enhances polymerization of actin, lumican (LUM) that is extracellular matrix protein, remained below the background level.

**Table 1:** DBS proteins with significantly different (*p*-value < 0.05) relative concentrations^**^between the three time points during 3 days dry immersion (DI) and background (2 days before DI).

**Protein**	**Gene**	***p*-Values**	**Background** **(Mean ±SD)**	**First day of DI** **(Mean ±SD)**	**Second day of DI** **(Mean ±SD)**	**Third day of DI** **(Mean ±SD)**	**Two days after DI** **(Mean ±SD)**
Serum albumin	ALB	0.019	4,534,033 ± 555,854	3,938,016 ± 764,137	3,960,933 ± 659,299	3,186,150 ± 402,832^*^	3,966,716 ± 651,992
Apolipoprotein A4	APOA4	0.027	126,956 ± 71,690	67,194 ± 41,250	54,624 ± 30,611^*^	42,357 ± 31,252^*^	89,280 ± 37,731
Angiotensinogen	AGT	0.046	72,563 ± 18,358	51,576 ± 8,891^*^	56,961 ± 30,298	38,088 ± 16,585^*^	67,992 ± 19,284
Hemopexin	HPX	0.025	184,660 ± 54,157	132,260 ± 56,296	110,542 ± 41,070^*^	89,996 ± 38,298^*^	137,266 ± 44,999
Bisphosphoglycerate mutase	BPGM	0.021	68,275 ± 60,399	58,244 ± 31,321	41,146 ± 10,969	129,953 ± 71,012	52,283 ± 25,731
Immunoglobulin heavy constant alpha 1	IGHA1	0.021	194,255 ± 42,435	151,775 ± 52,739	123,237 ± 43,118^*^	109,179 ± 48,846^*^	124,086 ± 30,872^*^
Proteasome subunit alpha type-2	PSMA2	0.030	9,528 ± 2,142	11,238 ± 2,685	15,998 ± 6,500^*^	9,925 ± 3,357	14,012 ± 2,639^*^
Transketolase	TKT	0.037	15,578 ± 7,274	24,559 ± 13,108	18,371 ± 7,727	30,221 ± 9,080^*^	16,935 ± 2,844
Phosphoglucomutase-2	PGM2	0.020	3,996 ± 2,215	6,258 ± 1,979	6,521 ± 1,588^*^	3,858 ± 1,513	7,120 ± 2,414^*^
Importin-9	IPO9	0.026	1,194 ± 220	1,316 ± 380	2,348 ± 839^*^	1,147 ± 767	1,224 ± 476
Lumican	LUM	0.011	12,078 ± 4,667	10,564 ± 2,619	6,078 ± 3,544^*^	5,317 ± 3,669^*^	6,838 ± 2,869^*^
Nucleosome assembly protein 1-like 4	NAP1L4	0.028	2,829 ± 1,767	2,065 ± 709	2,023 ± 917	1,264 ± 466	3,636 ± 1,162
Translin	TSN	0.017	564 ± 307	894 ± 580	1,652 ± 673^*^	442 ± 255	855 ± 563
Profilin-1	PFN1	0.030	2,904 ± 361	1,633 ± 776	420 ± 0^*^		878 ± 473^*^
NIF3-like protein 1	NIF3L1	0.039	1,168 ± 397	519 ± 203	1,890 ± 739	694 ± 17	1,221 ± 232
Exportin-2	CSE1L	0.032	630 ± 450	255 ± 158	188 ± 37	405 ± 148	1,815 ± 1,043
Carbonyl reductase (NADPH) 1	CBR1	0.043	920 ± 207	1,272 ± 707	1,789 ± 587^*^	708 ± 248	867 ± 425
Ras-related protein Rap-1b	RAP1B	0.039	6,920 ± 3,406	3,812 ± 2,846	9,128 ± 3,673	4,029 ± 2,010	7,560 ± 3,022
Myosin light chain 4	MYL4	0.011	906 ± 352	1,601 ± 902	1,386 ± 745	2,820 ± 1,050^*^	952 ± 262
Carboxymethylenebutenolidase homolog	CMBL	0.018	1,359 ± 565	842 ± 213	1,498 ± 374	606 ± 58	392 ± 113
NEDD8-activating enzyme E1 catalytic subunit	UBA3	0.008	527 ± 386	464 ± 47	1,799 ± 478	1,056 ± 100	800 ± 307
Thyroxine-binding globulin	SERPINA7	0.017	3,665 ± 618	3,076 ± 794	648 ± 218^*^	2,049 ± 1,157	4,010 ± 925
Zyxin	ZYX	0.043	804 ± 811	787 ± 699	1,462 ± 381	1,750 ± 236	532 ± 269
Aquaporin-1	AQP1	0.001	425 ± 285	734 ± 267	3,213 ± 0^*^	440 ± 29	355 ± 151

**Normalized intensity profile (sample area units).

* *Proteins with concentration significantly differed from the background values (2 days before DI) according to the results of post-hoc analysis*.

**Figure 1:**
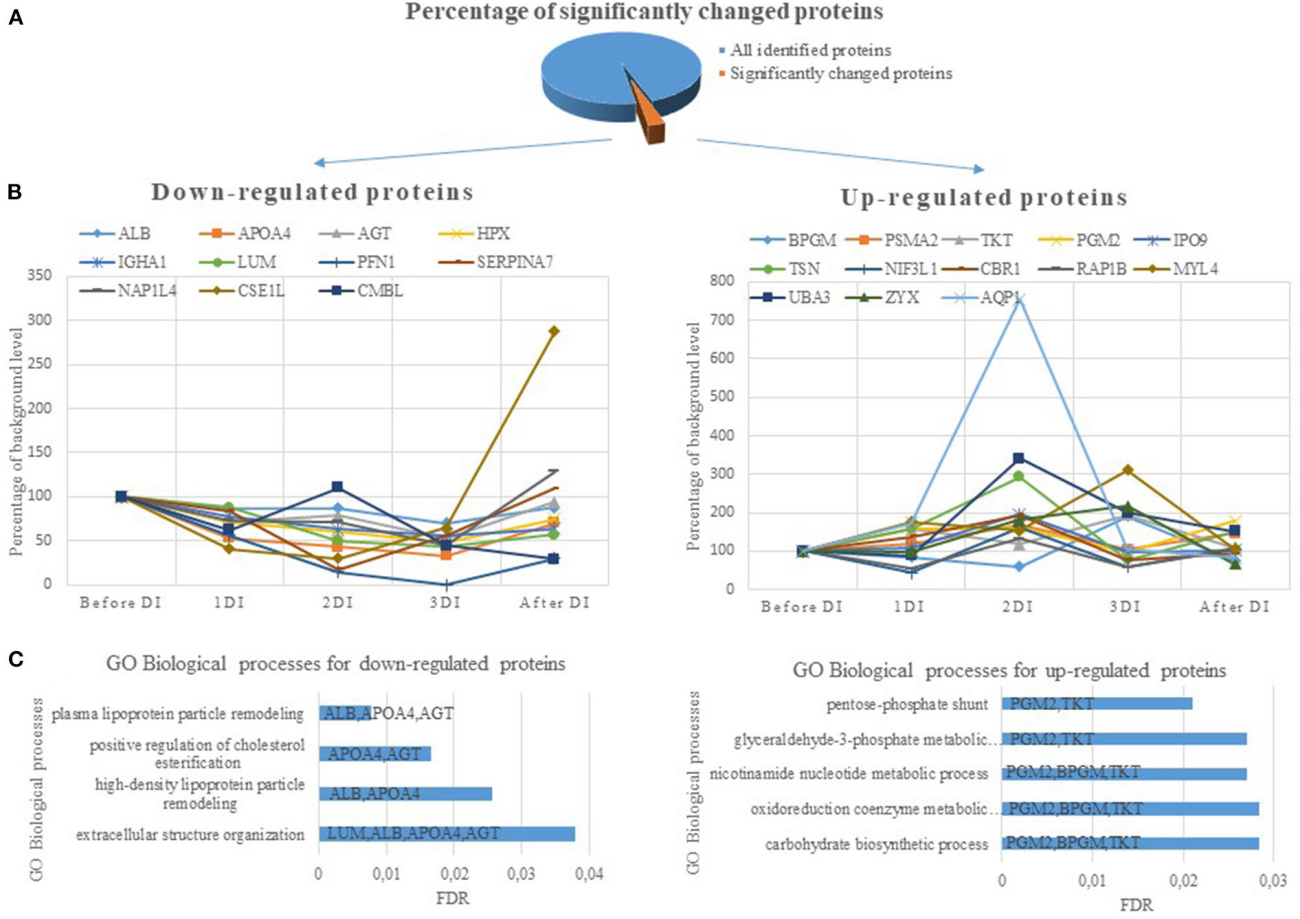
**(A)** The percentage of significantly changed proteins during 3 days dry immersion (DI). **(B)** The dynamics for up- and down-regulated proteins. The dynamics of changes in the protein content is indicated as a percentage of the background level (2 days before DI, the level at this experimental point is taken as 100%). **(C)** Biological processes involving DBS proteins with significantly different concentrations during 3 days DI. The value of the column means FDR (the smaller the FDR, the more reliable the process). The columns indicate the genes of proteins involved in this biological process.

The set of significantly changed proteins was divided into up-regulated and down-regulated proteins and analyzed using the String internet resource (Figure 1). According to the GO database, the following biological processes are distinguished for down-regulated proteins: processes associated with remodeling of plasma lipoproteins and organization of extracellular structures. For up-regulated proteins, biological processes associated with the pentose phosphate shunt have been identified. This reveals a cluster of proteins (PGM2, TKT, BPGM) (Figure 1) involved in different metabolic processes—carbohydrate biosynthetic process, nicotinamide nucleotide metabolic process, oxidoreduction coenzyme metabolic process. These proteins significantly increased on the second or third day of DI. Phosphoglucomutase-2 (PGM2) catalyzes the conversion of the nucleoside breakdown products ribose-1-phosphate and deoxyribose-1-phosphate to the corresponding 5-phosphopentoses. It also catalyzes the interconversion of glucose-1-phosphate and glucose-6-phosphate. Transketolase (TKT) like the PGM2 is also involved in glyceraldehyde-3-phosphate biosynthetic process. Bisphosphoglycerate mutase (BPGM) plays a key role in regulating hemoglobin oxygen affinity by controlling the levels of its allosteric effector 2,3-bisphosphoglycerate. These proteins are cytosolic proteins and they got into the analysis as a result of lysis of blood cells that were in a DBS samples. The most likely the main contribution to the change in the concentration of these proteins was made by erythrocytes, since there are the most active reactions of the pentose phosphate pathway along with the cytosol of liver cells, adipose tissue, and adrenal cortex. The pentose phosphate pathway of glucose oxidation is not associated with the formation of energy but provides anabolism of cells. In erythrocytes only NADPH is formed as a product of the pentose phosphate pathway. In this case pentose is not the final product as it is converted into phosphohexose which close the cycle or go into glycolysis completing the shunt. NADPH is an important component of antioxidant defense and it is necessary for the regeneration of glutathione which together with glutathione peroxidase destroys reactive oxygen species (ROS). Since NADPH is formed in erythrocytes only in pentose-phosphate shunt reactions an increase in the concentration of proteins of the pentose-phosphate shunt may be a response to oxidative stress.

Indeed physical inactivity was shown to enhance muscle ROS production (Lawler et al., 2003; Agostini et al., 2010) and to affect activity of antioxidant systems (Banerjee et al., 2003; Agostini et al., 2010). Experimental bed rest has been shown to be associated with oxidative stress and activation of the glutathione system (Dalla Libera et al., 2009; Agostini et al., 2010). Glutathione is one of the major antioxidant systems stimulated both at muscular and systemic level by activation of oxidative processes (Dobrowolny et al., 2008). Its action is principally mediated by a reaction catalyzed by glutathione peroxidase leading to oxidized glutathione disulfides (Lu, 2000). Glutathione concentration is particularly abundant in the liver and erythrocytes where it acts as local and systemic antioxidant agent (Qi et al., 2013). It's worth noting that countering oxidative stress can reduce muscle loss. Thus, antioxidant supplementation prevented disuse atrophy in animal models (Betters et al., 2004; Momken et al., 2011). There is a need to develop means to prevent oxidative stress during low physical activity. This is important because physical inactivity was shown to increase vascular superoxide production and impair endothelium-dependent vasorelaxation, which may contribute to endothelial dysfunction and atherosclerosis.

In general only blood cell cytosol proteins concentration increased while extracellular proteins (ALBU, APOA4, AGT, LUM, HPX, SERPINA7) decreased (Table 1; Figure 1) during DI and more strongly with each day of immersion. Such dynamics of proteins is possibly associated with a decrease in the synthesizing function of the liver. The data on the main site of synthesis of the aforementioned proteins lead to this conclusion. However, the influence of other factors on the change in the concentration of these proteins is not excluded. Thus a decrease in APOA4 may be associated with a decrease in high density lipoproteins which was detected after short (Leach, 1992) and long-term (Markin et al., 1998) space flights. Angiotensinogen reacted to the experimental conditions earlier than other proteins and a significant decrease in its concentration was observed on the first day. Angiotensinogen is a precursor of angiotensin and plays an important role in the renin-angiotensin system which is known to be involved in the adaptation of the body's water-electrolyte metabolism to the conditions of simulated microgravity (Gharib and Hughson, 1992). Therefore, a change in the concentration of angiotensinogen under DI conditions is natural and connected with redistribution of body fluids and changes in the volume of circulating blood. In addition a change in the concentration of aquaporin could be due to a decrease in the volume of circulating plasma. Aquaporin forms a water-specific channels in plasma membranes of red cells and thereby allowing water to move in the direction of an osmotic gradient. Indeed, numerous studies have shown that simulated microgravity affects aquaporins (Bu et al., 2012; Tamma et al., 2014).

The study of the physiological functions via DBS proteome analysis thus confirm the already established ideas about the physiological response to the DI conditions and provide new information about previously unknown mechanisms of the adaptive processes. Revealing of a new blood proteome specific changes due to extreme conditions including physical inactivity can identify potential targets for intervention and prevention of negative consequences.

As far as we know this is the first proteomics study concerning female response to simulated microgravity during 3-days DI. The DBS with capillary blood sampling was selected as a perspective sample collection technique which is less invasive and allows sampling with a greater frequency both in the ground based experiments and space flight conditions.

## Data Availability Statement

The datasets presented in this study can be found in online repositories. The names of the repository/repositories and accession number(s) can be found below: http://www.proteomexchange.org/, PXD027654.

## Ethics Statement

The studies involving human participants were reviewed and approved by Commission on Biomedical Ethics at the IBMP RAS. The patients/participants provided their written informed consent to participate in this study.

## Author Contributions

IL, VR, AK, and EN conceived and designed the experiments. OP and WS collected samples of dry blood spots. DK and WS performed sample preparation to mass-spectrometry. AK and AB conducted mass-spectrometric analysis. DK, AK, and LP wrote the article. All authors contributed to the article and approved the submitted version.

## Funding

This work was supported by the by Russian Science Foundation, grant # 21-74-20173.

## Conflict of Interest

The authors declare that the research was conducted in the absence of any commercial or financial relationships that could be construed as a potential conflict of interest.

## Publisher's Note

All claims expressed in this article are solely those of the authors and do not necessarily represent those of their affiliated organizations, or those of the publisher, the editors and the reviewers. Any product that may be evaluated in this article, or claim that may be made by its manufacturer, is not guaranteed or endorsed by the publisher.
